# Spatiotemporal analysis and environmental risk factors of visceral leishmaniasis in an urban setting in São Paulo State, Brazil

**DOI:** 10.1186/s13071-019-3496-6

**Published:** 2019-05-21

**Authors:** Luiz E. Prestes-Carneiro, Loris A. F. Daniel, Lívia C. Almeida, Lourdes Zampieri D’Andrea, André G. Vieira, Ivete R. Anjolete, Lenira André, Edilson F. Flores

**Affiliations:** 1 Infectious Diseases and Immunology Department, Universidade do Oeste Paulista, Presidente Prudente, São Paulo Brazil; 2 Center for Biomedical Sciences and Regional Laboratory, Instituto Adolfo Lutz, Presidente Prudente, São Paulo Brazil; 3 Statistics Department, School of Sciences and Technology, São Paulo State University, Presidente Prudente Campus, Presidente Prudente, São Paulo Brazil; 4 Municipal Secretariat of Environment, Presidente Prudente, São Paulo Brazil; 5 Supervision in Control of Endemics, Presidente Prudente, São Paulo Brazil

**Keywords:** *Lutzomyia longipalpis*, Entomological survey, Canine visceral leishmaniasis, Human visceral leishmaniasis

## Abstract

**Background:**

In Latin America, Brazil harbors the most cases of human visceral leishmaniasis (HVL). Since the early 1980s, the disease has spread to the urban centers of the north, and now the south and west of Brazil; it reached São Paulo state in the southeast in 1996, and Presidente Prudente in the western region in 2010. Our aim was to describe the spatiotemporal analysis and environmental risk factors associated with the dispersion of VL in Presidente Prudente, an urban setting with recent transmission.

**Methods:**

An entomological survey was carried out from 2009 to 2015. A canine visceral leishmaniasis (CVL) serosurvey was performed from 2010 to 2015 using enzyme-linked immunosorbent assays (ELISA), a dual-path platform CVL rapid test, and indirect fluorescent antibodies (IFAT). Data from HVL cases were obtained from the Municipal Surveillance Epidemiology Center from 2013 to 2017. Data on water drainage and forest fragments were obtained from public platforms and irregular solid-waste deposits were determined by monthly inspections of the urban area. Kernel density maps of the distribution of CVL were constructed.

**Results:**

From 2009 to 2015, *Lutzomyia longipalpis* sand flies were found in all seven areas of Presidente Prudente. From 2010 to 2015, 40,309 dogs were serologically screened and 638 showed positive results, i.e. a prevalence rate of 1.6%. From 2013 to 2017, six human cases were diagnosed with a mortality rate of 33.3%. In 2015, 56 points of irregular solid-waste deposits were identified, predominantly in the neighborhoods. Three different hotspots of CVL showed an increased distribution of vectors, seropositive dogs, irregular solid-waste deposits, forest fragments and water drainage.

**Conclusions:**

The use of tools that analyze the spatial distribution of vectors, canine and human VL as environmental risk factors were essential to identifying the areas most vulnerable to the spread or maintenance of VL. The results may help public health authorities in planning prevention and control measures to avoid expansion and future outbreaks.

## Background

In many countries, visceral leishmaniasis (VL) is considered an endemic zoonosis and a public health concern. Brazil currently harbors 96% of the cases in Latin America [1, 2]. Since the early 1980s, VL has spread to the urban centers of the north and, more recently, the south and west of Brazil, with cases reported in humans or dogs in 26 of the 27 states [3, 4]. In São Paulo state, the richest and most developed state in Brazil, VL has spread geographically. Since the vector *Lutzomyia longipalpis* was found in Araçatuba in 1996, 193 of the 645 municipalities (29.9%) have reported its presence (as of January 2018). From 1997, when canine visceral leishmaniasis (CVL) was first identified, until 2014, 108 of the 645 municipalities (16.7%) reported cases of CVL. From 1999 to 2017, 97 municipalities (15.0%) reported autochthonous cases of human visceral leishmaniasis (HVL) with 2836 autochthonous HVL cases confirmed and 242 deaths, presenting a mortality rate of 8.5% [5–7]. Regional Network for Health Assistance 11 (RNHA 11) is composed of 45 municipalities and corresponds to the Brazilian Institute of Geography and Statistics (IBGE) mesoregion 8, the western region of São Paulo state. This region is experiencing a rapid spread of VL.

Previously, in the state of São Paulo, the disease was known only through imported cases. In 1996, the finding of the vector *Lu. longipalpis* in Araçatuba, 170 km from Presidente Prudente, triggered the implementation of the Visceral Leishmaniasis Control and Surveillance Programme (VLCSP) of São Paulo state [8, 9]. Actions referring to the disease, vector and reservoir were defined, and in 1999 they were implemented in the region of Presidente Prudente [8]. Presidente Prudente, the largest and most important city in RNHA 11, has been proposed as a connection axis between different states of Brazil, including Mato Grosso do Sul and Paraná states, and a route for the spread of VL [9, 10]. Due to the proximity of Araçatuba, where sand flies have been detected since 1996, an entomological survey was initiated in 1999 in Presidente Prudente, and in 2009, *Lu. longipalpis* sand flies were found close to the downtown area. The dispersion routes of CVL were established by allochthonous dogs coming from surrounding endemic counties in the western region. They were found in virtually every area of the city. In 2010, autochthonous CVL was detected in different urban settings [9].

The environment plays a key role in the life-cycle and dispersion of VL. Environmental factors such as extensive interconnection of roads from small towns and districts to mid-sized cities favor a daily flow of people, cars, animals and goods [9, 11]. Between 1990 and 2010 the rainforest was replaced by pasture and sugar monoculture, facilitated by the influx of a large number of migrants, rural workers engaged in sugarcane plantation and cutting, coming from VL endemic areas such as Minas Gerais and northeast Brazil [9, 12]. Large artificial lakes constructed in the western region as natural recreational areas also have a role in the change of temperature, humidity and precipitation [13]. Taken together, it has been proposed that these factors have intensified the spread of infection through the western region of São Paulo state [7–10]. However, both throughout the state and at the regional level, there are many questions that have not yet been sufficiently addressed. (i) Are other vectors involved in transmission in the municipalities where dogs and humans were infected but *Lu. longipalpis* was not found? (ii) Although diseases spread predominantly by contiguity with adjacent foci, what is the impact of an extensive network of highways on the scattered disease areas? (iii) Even with regular campaigns to stimulate knowledge, attitudes and practices on the importance of cleaning gardens, yards and vacant household lots and the correct disposal of household and solid waste, why is the population remaining indifferent to these measures?

In the Americas, domestic dogs are the main reservoir of *Leishmania infantum* (syn. *Leishmania chagasi*). As a result of large numbers of parasites in their skin and viscera, dogs facilitate blood-meal acquisition and parasite infection in the vectors. Some infected dogs remain asymptomatic for inconsistent periods of time and do not develop clinical signs or symptoms [14]. In middle-sized and large Brazilian cities, stray dogs roam freely, mainly in the poor areas and shanty towns on the periphery, helping vectors to thrive and increasing the risk of CVL dispersion [14, 15]. In epidemiological studies of vector-borne diseases, geospatial analysis has been used to identify temporal and spatial dispersion of disease and to predict the influence of the neighborhood infected areas on spreading the disease to other suitable areas [16].

In São Paulo state, similar to other regions countrywide, despite all the measures taken by Brazilian public health agencies in the control of VL, the spread of the disease has expanded geographically. Our aim was to uncover spatiotemporal patterns of CVL occurrence and to associate these patterns with environmental risk factors that can promote the dispersion of VL in Presidente Prudente, an urban setting with recent transmission and few reported cases of HVL.

## Methods

### Study design and setting

**Fig. 1:**
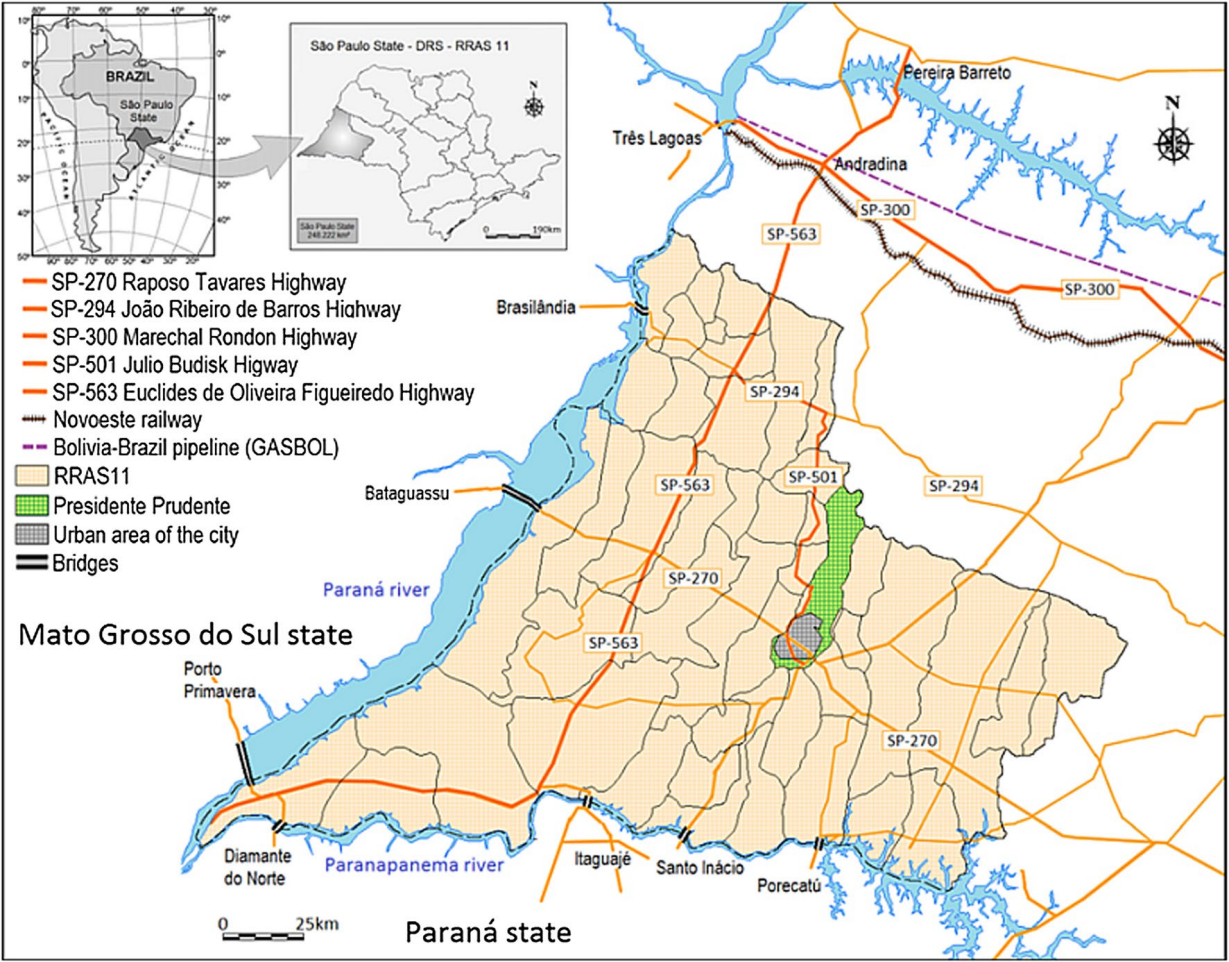
Location of Presidente Prudente in RNHA 11 of São Paulo state and road connection network. This map shows the connection between the municipality of Presidente Prudente and well-known endemic regions such as Mato Grosso do Sul State and the municipalities of Andradina and Dracena, in São Paulo state. Extensive highways link the VL endemic regions of Mato Grosso do Sul and São Paulo states to Presidente Prudente and from Presidente Prudente to the capital São Paulo and Paraná state

A cross-sectional study was conducted between January 2009 and December 2017 in the city of Presidente Prudente, in the western region of São Paulo state, Brazil (22°07′32″S, 51°23′20″W). The state of São Paulo is divided into 15 mesoregions, which are subdivided further into microregions. Presidente Prudente is located in mesoregion 8 (Fig. 1). The municipality has a typical tropical climate with a dry winter and wet summer and an average annual temperature of 23.5 °C. The city is a mid-sized urban center about 560 km from the state capital, São Paulo. According to the census by the IBGE, in 2016 the estimated population was 223,749 inhabitants, with 214,799 (96%) living in the urban area. The municipality covers an area of 562,107 km^2^, and the urban area covers 16.56 km^2^ [17]. The city of Presidente Prudente is considered a crossroads connecting São Paulo, the capital city, and other states such as Mato Grosso do Sul, Paraná and Minas Gerais and is considered an epidemiological route of the disease. The 45 municipalities that comprise RNHA 11 are under the supervision of Presidente Prudente. The region has an estimated population of 900,000 inhabitants. In 2016, the canine population was estimated to be 41,604 animals, calculated based on a ratio of 2 dogs per 10.7 inhabitants, according to the Center for Zoonosis Control (CZC) in Presidente Prudente. Control actions of the Visceral Leishmaniasis Control and Surveillance Programme (VLCSP) [8] have been in place since 2010.

Archival databases were obtained from Brazilian public health agencies and are available to download upon formal request: Supervision in Control of Epidemic (SUCEN), http://www.saude.sp.gov.br/sucen-superintendencia-de-controle-de-endemias; Center of Regional Laboratory of the Adolfo Lutz Institute of Presidente Prudente V (CRL-CBSALI-PPV), http://www.ial.sp.gov.br/ial/o-ial/quem-e-quem/centro-de-laboratorio-regionais; National System on Diseases Notification (SINAN), http://portalsinan.saude.gov.br/; São Paulo Epidemiological Bulletin (BEPA), http://www.saude.sp.gov.br/ses/perfil/profissional-da-saude/informacoes-de-saude-/boletim-epidemiologico-paulista-bepa; Epidemiological Surveillance Center (CVE-SP), http://www.saude.sp.gov.br/cve-centro-de-vigilancia-epidemiologica-prof.-alexandre-vranjac/; and Municipal Surveillance Epidemiology Center, http://www.presidenteprudente.sp.gov.br/site/unidades/sms_vigilancia_epidemiologica.xhtml. Current shapefile databases and base maps were downloaded from the IBGE website.

### Entomological survey

**Fig. 2:**
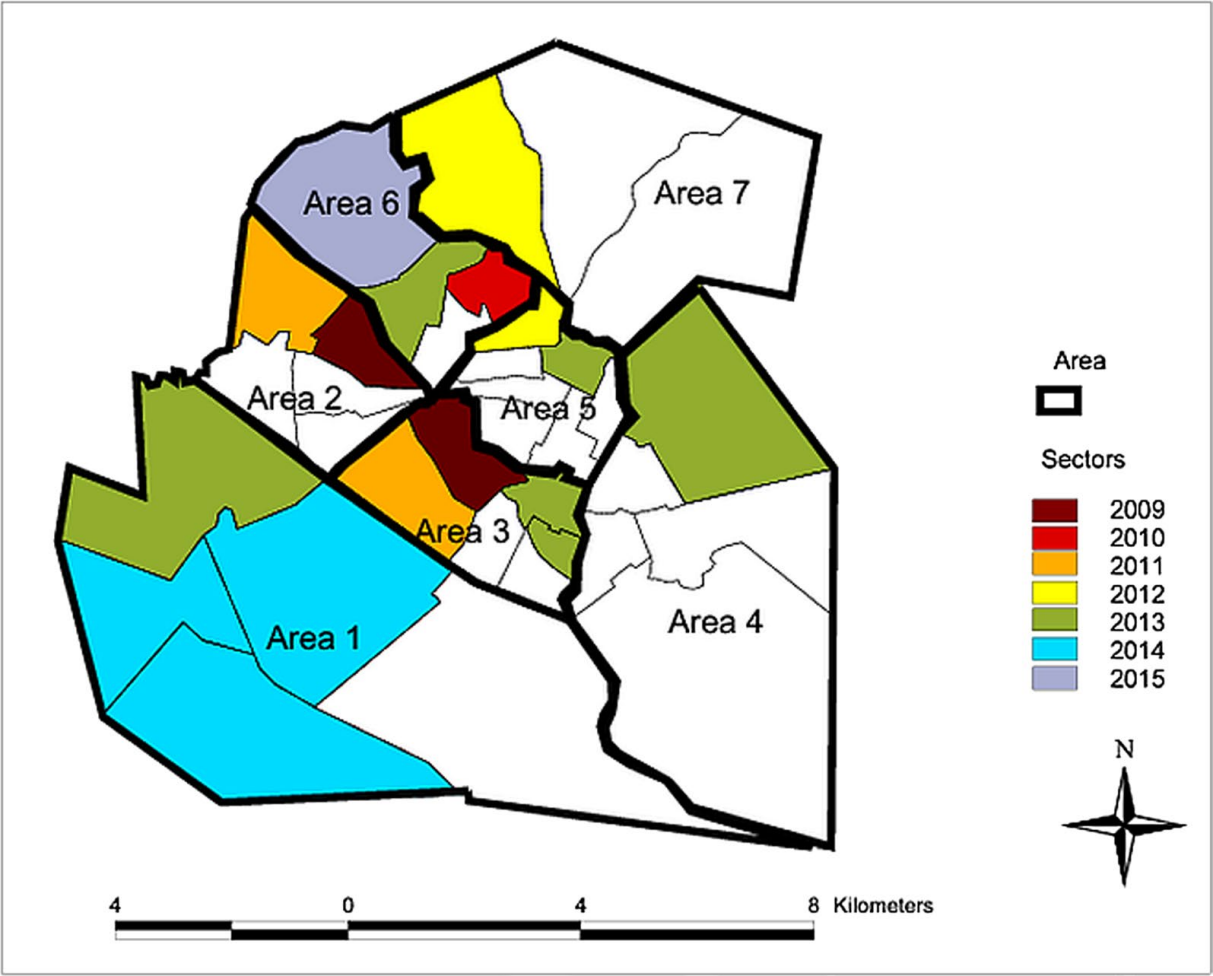
Spatiotemporal findings for *Lu. longipalpis* in the urban area of Presidente Prudente, 2009 to 2015. The entomological surveys and database collection for public health programmes use a specific division of the urban area. The city of Presidente Prudente is divided into sectors that represent a number of households to be visited by one or two health agents in the field. The areas are composed of up to five connecting sectors presenting similar characteristics of blocks/households. This division is used for epidemiological surveys of diseases, including dengue fever, CVL and HVL. In São Paulo state, delimitation of sectors and areas was created by the State Health Secretary (Source: SUCEN)

The city of Presidente Prudente covers seven areas with regard to epidemiological diseases, including dengue fever, CVL and HVL. The areas are divided into sectors (Fig. 2, Table 1). Entomological surveys are overseen by the Visceral Leishmaniasis Control Programme of São Paulo state (VLCPSP) [8], aiming to monitor the distribution of *Lu. longipalpis* in non-vulnerable, non-receptive silent municipalities, as defined by the absence of confirmed autochthonous cases of HVL and CVL, without the presence of the vector. In these municipalities, an entomological survey is recommended to detect the presence of *Lu. longipalpis* and provide information on its distribution, allowing identification of the risk areas where control measures should be intensified.

**Table 1:** Spatiotemporal occurrence of *Lu. longipalpis* in the urban area of Presidente Prudente, São Paulo state, Brazil from 2009 to 2015

Date	Area	Sector	No. of traps	No. of times	No. of sand flies	Species
Feb 2009	3	1	10	10	M:689; F:67/M:1; F:2	*Lu. longipalpis/E. cortelezzi*
Mar 2009	2	3	9	6	M:3	*Lu. longipalpis*
Sep 2010	6	3	16	16	M:1	*Lu. longipalpis*
Mar 2011	2	1	1	1	M:4; F:1	*Lu. longipalpis*
Mar 2011	3	2	23	23	F:2	*Lu. longipalpis*
Feb 2012	7	1	2	2	M:1	*Lu. longipalpis*
Apr 2012	5	1	22	9	M:7; F:3	*Lu. longipalpis*
Feb 2013	6	2	24	15	M:1; F:1	*Lu. longipalpis*
May 2013	3	3	2	2	M:1	*Lu. longipalpis*
Jul 2013	5	5	20	20	M:1; F:4/F:1	*Lu. longipalpis/Brumptomyia brumpti*
Sep 2013	1	1	2	2	M:1	*Lu. longipalpis*
Sep 2013	4	1	4	4	M:1	*Lu. longipalpis*
Dec 2013	3	5	4	1	M:1	*Lu. longipalpis*
Jan 2014	1	3	13	4	M:3	*Lu. longipalpis*
Jan 2014	1	4	40	13	M:1; F:3	*Lu. longipalpis*
Nov 2014	1	2	30	39	M:2	*Lu. longipalpis*
Jan 2015	6	1	3	2	M:2	*Lu. longipalpis*
Total			215	169	804 M:720; F:84	

*Abbreviations*: F, female; M, male

In the urban area of Presidente Prudente and in some districts, the entomological survey began in 1999 during the most favorable periods for collecting the vector, from October to January and from February to April, and extended through May as needed. To standardize collections, the number (ranging from 1 to 40) and frequency (ranging from 1 to 39) of CDC light traps (Horst Ltd, São Paulo, Brazil) installed varied according to the density and infestation of sand flies [8, 18]. The traps were placed approximately 1 m from the ground in places susceptible to the presence of phlebotomines and/or in the shelters of domestic animals, with an average temperature above 20 °C and relative humidity > 70%. In each sector of the different areas, four properties with risk factors for the presence of phlebotomines were selected, including a large peri-domicile, abundant vegetation, accumulation of organic matter in the soil and the presence of domestic animals from which the female sand fly can obtain a potentially infected blood meal. The traps were operated between 17:00 and 07:00 h. Four traps were installed for three nights in each sector. After capture, nylon-mesh cages were placed in plastic bags, tagged and refrigerated (1–7 °C) until identification was performed by the Regional Entomology Laboratories of SUCEN, Coordination of Disease Control, Secretary of State of Health. The collected sand flies were processed and were identified using the taxonomic key of Galati [19]. In 1999, when the survey started in the urban area of Presidente Prudente, the traps were installed in seven areas concomitantly. When the vector was found in a particular area or sector, the survey ended in this setting. In 2015, after the vector was found in all areas, the entomology survey ended.

### Canine visceral leishmaniasis survey

Canine selection took place in all areas and sectors (Fig. 2, Table 1) of the city from January 2010 to December 2015, by either passive or house-to-house survey conducted by the CZC [9]. Briefly, an active house-to-house search was conducted when *Lu. longipalpis* was found in a particular area of the city or every time a dog was diagnosed with CVL; immediately, all households within a radius of 200 m were visited and the dogs were tested for CVL. A passive survey was conducted when a dog’s owner found the symptoms described by the CZC as suspicious for CVL as previously published [9].

We used data from 40,309 domiciled dogs of different ages, submitted to a serum survey, using antibody tests to detect *Leishmania*, according to the Brazilian Ministry of Health (MoH) and VLCPSP guidelines [8]. In Presidente Prudente, the Center of Regional Laboratory of the Adolfo Lutz Institute (CRL-CBSALI-PPV) is responsible for the diagnostic tests for CVL. The VLCSP supervises the main actions to reduce morbidity and mortality, aimed at early diagnosis and treatment of human cases, vector control and identification and euthanasia of seropositive domestic dogs [8]. In 2009, the screening and confirmatory tests adopted by the MoH for CVL serological surveys were enzyme-linked immunosorbent assays (ELISAs), produced by Bio-Manguinhos/Fiocruz, MoH, Rio de Janeiro, Brazil. For ELISA-positive samples, a confirmatory test using indirect fluorescent antibodies (IFAT), produced by Bio-Manguinhos/Fiocruz, was performed [9, 20]. The ELISA assay for CVL consisted of the reaction of sera from dogs with soluble and purified *Leishmania major* antigens obtained from culture *in vitro*. The sensitivity of the ELISA-CVL Bio-Manguinhos/Fiocruz was previously determined by two different authors to be 72 and 100% and the specificity was 87.5 and 96.6%, respectively [21, 22]. The IFAT test consisted of the reaction of sera of dogs with parasites (*Leishmania*), fixed on microscopic slides. The sensitivity of the IFAT-CVL Bio-Manguinhos/Fiocruz was previously determined by the same authors to be 68.0 and 100% and the specificity was 87.5 and 96.6%, respectively [21, 22]. From December 2011 to the present, the MoH replaced ELISA/IFAT tests with a new protocol using a dual-path platform (DPP) CVL rapid test, produced by Bio-Manguinhos/Fiocruz, and ELISA-CVL as a confirmatory test [20]. The DPP utilizes a recombinant protein K39 (rK39) antigen, a 39 amino acid sequence of a cloned specific kinase region of *Leishmania infantum*, which is widely used in the diagnosis of CVL. For the immunochromatographic assay, rK39 was shown to have a sensitivity of 91.5% and specificity of 94.7% in a previous study [23]. Animals with positive samples in the screening test (ELISA from January 2009 to November 2011 and DPP CVL rapid test from December 2011 until the present) and a negative result in the confirmatory test (IFAT and ELISA, respectively) were re-sampled. Only concordant samples that were positive in the screening test and positive in the confirmatory test were considered seropositive [9].

### Human visceral leishmaniasis

Cases of HVL were confirmed by clinical/epidemiological criteria and laboratory diagnostics according to the Manual of Surveillance and Control of Visceral Leishmaniasis of São Paulo state from January 2013 to December 2017 [24]. The data collection process was performed by the Municipal Epidemiological Surveillance, in which confidentiality was assured.

### Irregular disposal of solid waste, water drainage and forest fragments

Municipal solid waste is defined as the remains of human activities in urban areas and is considered undesirable when the municipality does not have a well-developed waste recycling system. The deposit area covers an area > 4 m^2^ with a minimum height of 30 cm. The deposits are usually in a solid state [25]. In this study, residential, commercial or industrial waste, or waste produced by public cleaning was considered. From January to December 2015, inspections were carried out monthly in the streets of the city of Presidente Prudente by the Mayor’s office. To support the environmental analysis, a map of the urban area of Presidente Prudente, delineating hydrography and forest fragments was created using the “shapes” and base maps from IBGE. In addition, a forest fragment map was created using satellite images in which the fragments were defined as areas with vegetation surrounded by anthropogenic or natural barriers capable of significantly decreasing the flow of animals and pollen and seed dispersion. The analysis was performed with ArcGIS (ESRI, Redlands, CA) geographical information system software, using the Universal Transverse Mercator coordinate system (UTM) and Datum SIRGAS 2000 as a reference, with a scale of 1:100,000 [26].

Cases of HVL and CVL were added in the context of the analysis of point processes (events) from the geographical coordinates representing the exact locations of the households with positive humans and dogs. In this study, we did not break the confidentiality of the households; the databases were masked using point-pattern analysis only. The objective of this analysis was to study if the spatial distribution of these points presented clusters and not simply random or regular events [27]. The kernel density estimation is a two-dimensional function that considers events within a region (defined by a radius), counting the points contained therein, indicating the area of greater or lesser concentration of the events being analyzed to generate a grid in which each cell presents the value of the intensity, density, ratio between attributes, etc.

In the simplest case, where each point corresponds only to the occurrence of the event, it is an intensity estimator. If *s* is an arbitrary location in region *R* and *s*1, *s*2, *s*3, …, *sn* are locations of *n* observed events, then the intensity in *s* is estimated by a fourth-order function whose intensity estimator is expressed as

$$\hat{\lambda }_{\tau } (s) = \sum\limits_{{h_{i} \le \tau }} {\frac{3}{{\pi \tau^{2} }}\left( {1 - \frac{{h_{i}^{2} }}{{\tau^{2} }}} \right)^{2} }$$

In this study, a two-dimensional function of the positive serum samples was created to provide a proportional value of the intensity of samples per unit area. With this function, it is possible to count all the points within a region of influence, weighting them by the distance of each one from the location of interest. In three different sites, based on the presence of increased numbers of infected dogs, small rivers, forest fragments and irregular solid waste deposits, environmental risk factors that favor the development and maintenance of sand flies, a sphere of influence of 1.2 km was established (represented by circles). The density results were stored in a raster file in grid format. Ten-meter pixels were set as the spatial resolution using a scale of 1:1200 m.

### Statistical analysis

The average age and the proportion (95% confidence limits) of individuals infected with VL were estimated. To estimate the annual incidence of cases, the direct method (Wilsonʼs method [28]) was used with confidence interval estimates.

## Results

### Potential dispersion routes of infection: the Presidente Prudente hub

Presidente Prudente is linked by an extensive network of highways. The Marechal Candido Rondon (SP 300) links two VL endemic regions: Três Lagoas, on the border of Mato Grosso do Sul state, and Araçatuba and Bauru in the northeastern region of São Paulo state, approximately 106 miles apart (Fig. 1). The Euclides Figueiredo highway (SP 563) links the VL endemic regions of Andradina and Dracena, approximately 72 miles from Presidente Prudente. The Julio Budisky highway (SP 501), in conjunction with João Ribeiro de Barros (SP 294), links Dracena to Presidente Prudente. This transport network could promote vector invasion and pathogen transport, creating new endemic areas. Coming from Campo Grande, Mato Grosso do Sul state, SP 270 Raposo Tavares passes through Presidente Prudente toward the capital, São Paulo.

### Entomological survey and spatiotemporal occurrence of *Lu. longipalpis* in the urban area of Presidente Prudente

**Fig. 3:**
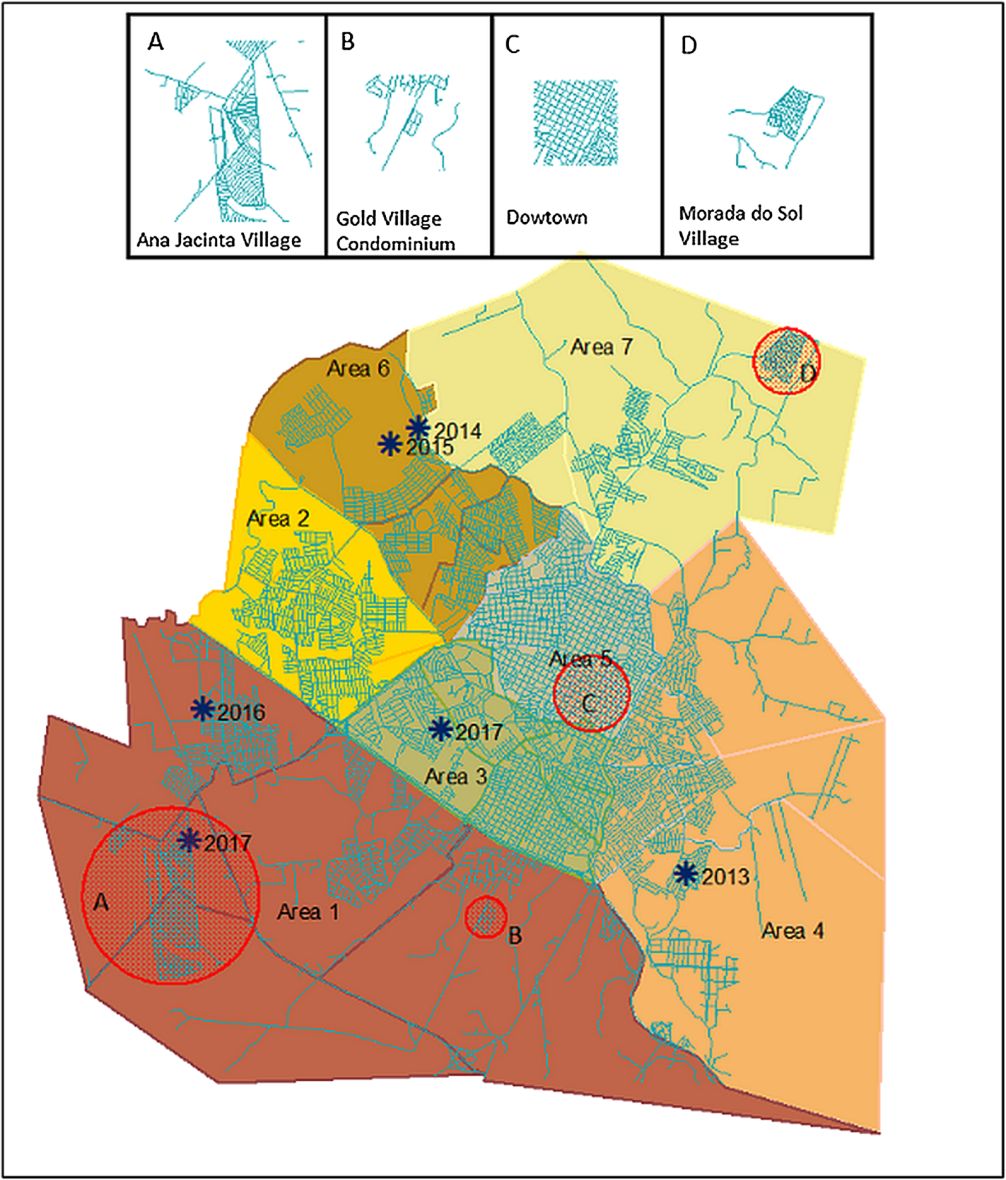
Geolocation of the individuals infected with VL in the urban area of Presidente Prudente. The circles represent the location of their residence. A, B, C and D at the top of the figure represent different urban features of the city. A, B and D represents areas on the outskirts of Presidente Prudente. *The location of the residence of VL infected individuals

From 2009, when the first sand flies were found, to 2015, 804 insects (720 males and 84 females) belonging to three species were collected: *Lutzomyia longipalpis*, *Evandromyia cortelezzii*, and *Brumptomyia brumpti*. The areas are divided into sectors and the sectors into blocks (Table 1, Fig. 2). The first sand flies were captured from 2009 to 2012 in sectors close to the downtown area. After 2012, sand flies were found in sectors farthest from the downtown area (Table 1, Figs. 2, 3, area C). In area 3, increasing numbers of sand flies were found in traps placed on 3 consecutive nights next to a chicken coop and one kennel (Table 1). In 2013, sand flies were found in three different areas on the periphery: areas 6, 1 and 4 which were 8.8, 10.5 and 6.0 km from the downtown area, respectively. In Presidente Prudente, *Lu. longipalpis* has spread from sectors close to downtown to the periphery (Table 1, Figs. 2, 3, area C). There was a great variation not only in the number of traps, varying from 1 to 40, but also how many times the traps were installed, varying from 1 to 39 times (Table 1).

### Spatiotemporal distribution of seropositive dogs from 2010 to 2015

**Fig. 4:**
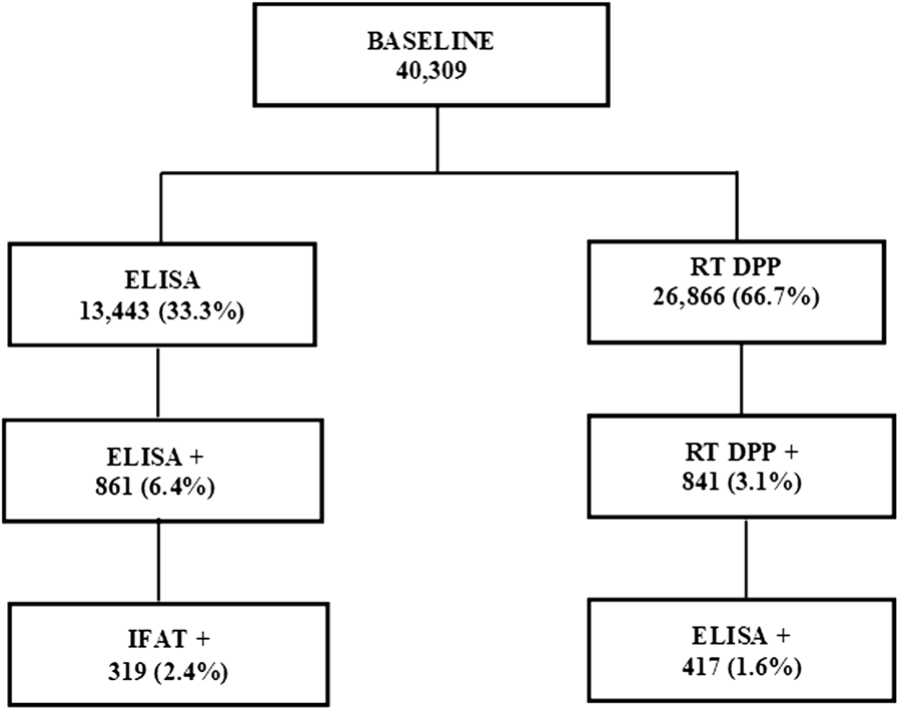
Seroprevalence of canine visceral leishmaniasis. Procedure framework designed for the serum survey in Presidente Prudente. Baseline: 40,309 dogs were surveyed from 2010 to 2015. The dogs were tested according to MoH recommendations using enzyme-linked immunosorbent assay (ELISA), indirect fluorescent antibodies (IFAT) and the rapid test dual-path platform

From 2010 to 2015, 40,309 dogs were serologically screened and 638 (1.6%) were found to be positive for CVL; 8002 (19.9%) were screened from 2010 to 2011, 20,650 (51.2%) from 2012 to 2013, and 11,657 (28.9%) from 2014 to 2015 (Fig. 4).

**Fig. 5:**
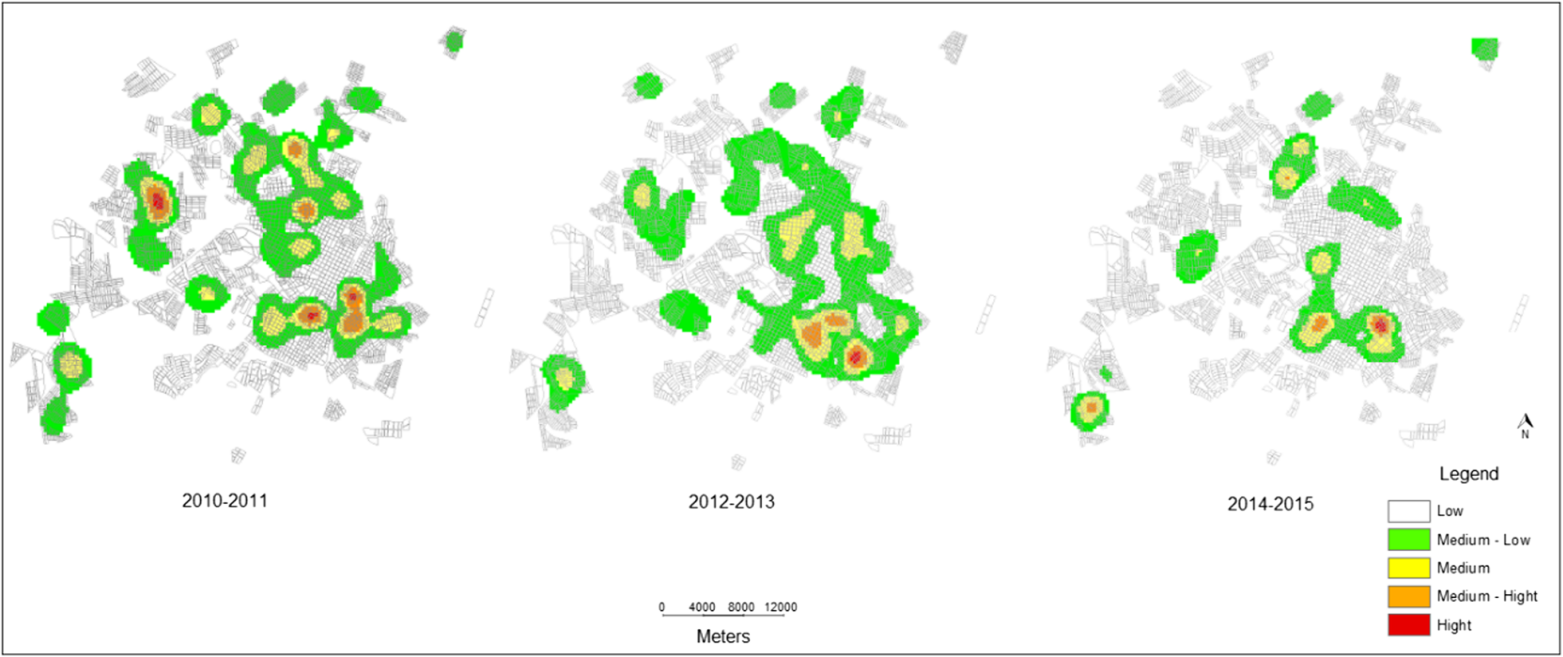
Kernel density estimation of CVL seropositive dogs. The hotspots represent areas where a higher density of seropositive dogs was observed, from 2010 to 2015. The data were condensed over 2-year periods. In the map legend, the lighter colors represent low density of CVL cases and the darker colors represent the presence of hotspots

The spatiotemporal distribution of VL seropositive dogs was irregular throughout the analysis period, with specific hotspots in vulnerable settings remaining (Fig. 5). In the period 2010–2011, 296 (40.2%) dogs were seropositive for CVL, and increased levels of seropositive dogs, demonstrated by hotspots on the kernel map, were found in three different areas of the city. The eastern zone (area 4) showed three different hotspots, the northern zone (area 3) showed two hotspots, and one hotspot was found in the western zone (area 1) (Figs. 2, 5). In the second two-year period, 2012–2013, 117 (15.9%) seropositive dogs were identified. The intensity demonstrated by the kernel map showed that high levels of seropositive dogs were maintained in the eastern zone (area 4), with three different hotspots. In the third two-year period, 2014–2015, a higher number of seropositive dogs, 323 (43.9%), was identified and according to the kernel map, a new hotspot was found in the southwest region (area 1). There was no statistical significance in the number of positive dogs in the last biennium compared with the first biennium.

### Cases of human visceral leishmaniasis, 2013–2017

The first autochthonous case of HVL was identified in 2013 in the eastern area, area 4, on the outskirts of Presidente Prudente (Fig. 3), where a high number of cases of CVL were found throughout the period. In 2014 and 2015, two patients were identified in area 6, also a region where a high number of cases of CVL were found. In 2016 and 2017, two infected individuals were found in area 1 on the outskirts, and in 2017 another infected individual was found in area 5, in a sector near downtown. From 2013 to 2017, six patients, distributed not only on the periphery but also in regions near the downtown area, were diagnosed and treated in the Regional Hospital of Presidente Prudente; they ranged in age from 1.8 to 75 years (mean, 34.62 years; 95% confidence interval, CI: 3.32–65.91) (Fig. 3). Two patients from the city of Presidente Prudente have died so far (mortality rate, 33.3%). An average of 1.2 cases/year were observed (incidence = 0.000579%, 95% CI: 0.000115–0.002890%).

### Kernel intensity of infected dogs overlapping with solid-waste deposits, forest fragments and water drainage areas: 2009–2015

**Fig. 6:**
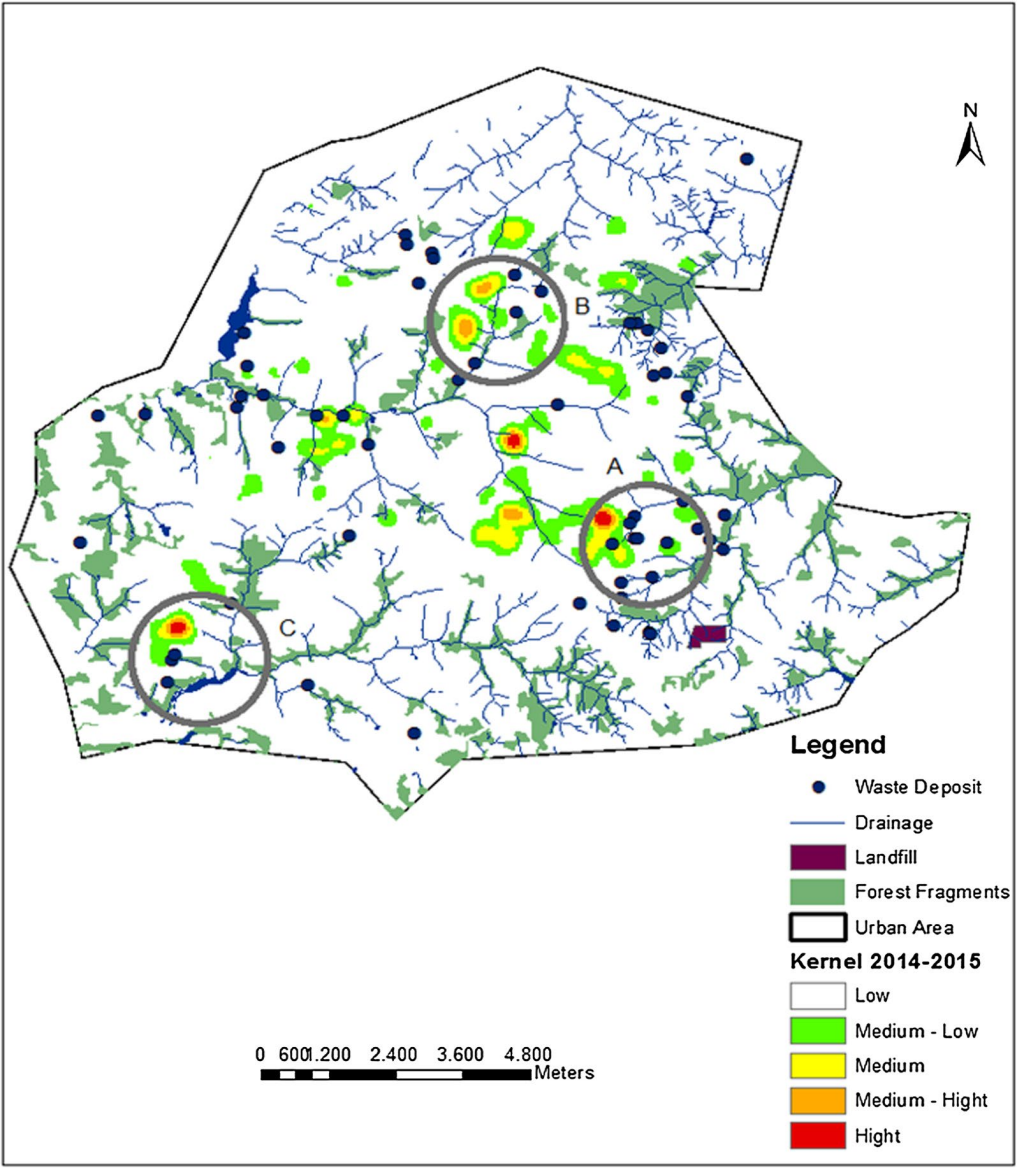
Kernel density of infected dogs overlapping with solid-waste deposits, forest fragments and water drainage areas. This map represents overlapping of CVL serological titres (kernel density), image classification of the forest fragments (red spots), drainage systems formed by streams and rivers in the city (blue linear features) and irregular deposits of solid waste (blue dots) in the urban area of Presidente Prudente, São Paulo state, Brazil. Circles (A, B and C) represent clusters with the co-presence of seropositive dogs, forest fragments, active drainage system and irregular deposits of solid waste. A buffer of 1200 m radius was used as a simulation of the sand fly flight range (Gaussian function and smooth kernel of 1200 m radius)

**Fig. 7:**
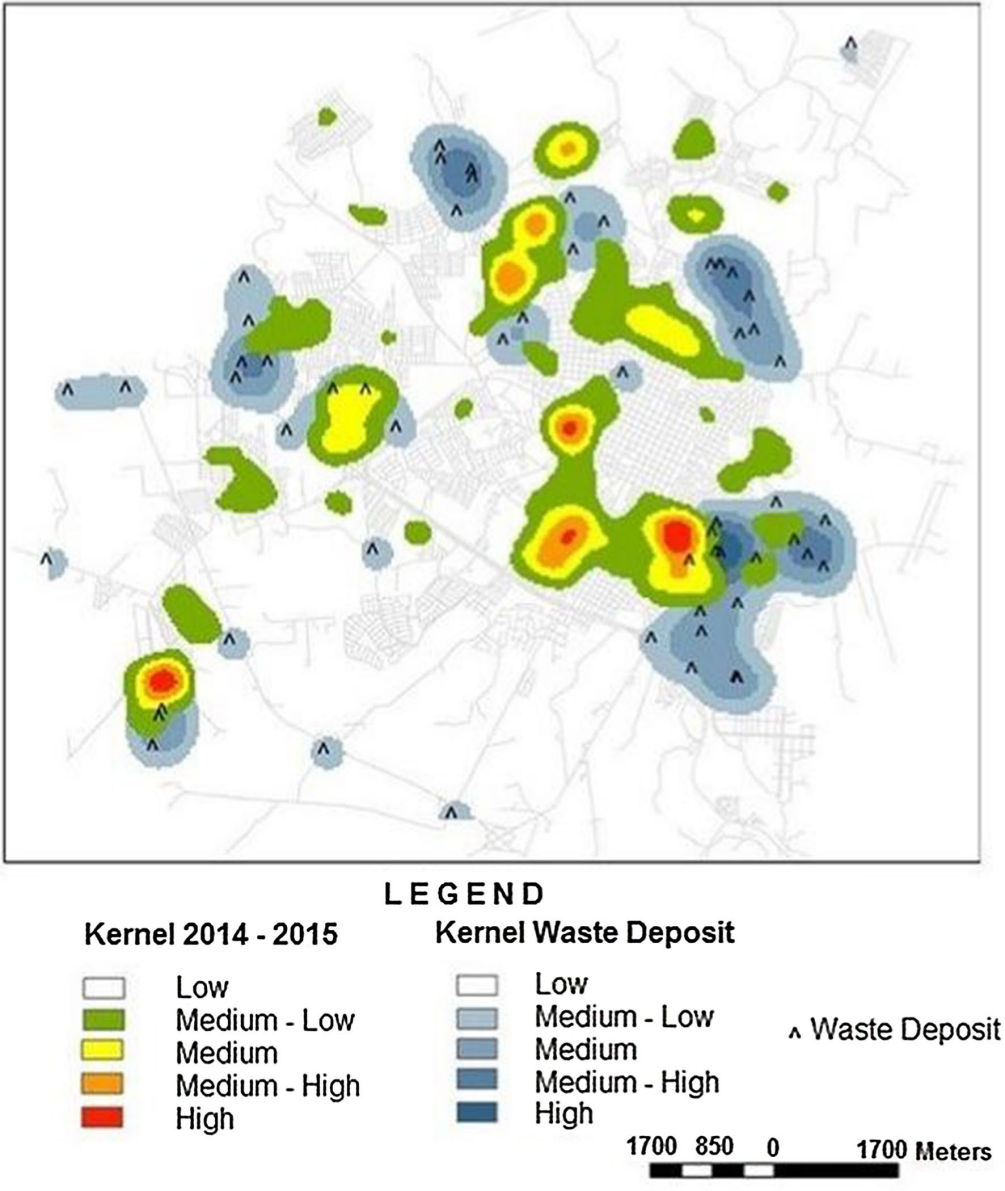
Kernel density of infected dogs and irregular solid waste deposits. This map represents the hotspots of CVL in the biennium 2014–2015 and the 56 points of irregular solid waste deposits. In 2015, inspections were carried out monthly in the streets of the city of Presidente Prudente by the Mayor’s office

Irregular solid-waste deposits were found in 56 locations in the urban area of Presidente Prudente, from residential, civil construction, sweeping, commercial and industrial plants. These were found especially along the river basin. Increased numbers of infected dogs (hotspots), solid-waste deposits and forest fragments were present in all the vulnerable areas (Fig. 6): clusters A in the eastern zone, B in the northern zone and C in the western zone; and in area 4, where the public landfill is located. Figure 7 shows the kernel density of infected dogs from the biennium 2104–2015 and the points of the solid waste deposits determined in 2015. It was demonstrated that most of the irregular solid waste deposits are located next to the CVL hotspots, not only in the periphery but also in the central area. No correlation was found between CVL hotspots and irregular solid waste deposits. The hydrographic basin of the urbanized area mainly consists of four rivers: Cascata and Gramado rivers in the northern and eastern zones, Limoeiro river in the western zone and Cedro river in the southeastern zone, associated mostly with forest fragments. In areas where the hydrographic basin does not include forest fragments, artificial canals had been constructed previously. In the urban area of Presidente Prudente, several residential villages and private condominiums have been constructed on the outskirts, far from the downtown area and neighborhoods (Fig. 3, areas A, B and D). There was no statistical significance associated with the clusters of seropositive dogs, forest fragments, hydrography and irregular disposal of solid waste in relation to potential areas where vectors were present (circles A, B and C).

## Discussion

Leishmaniasis is ranked as second in mortality and fourth in loss of disability-adjusted life years among tropical diseases, and it is not receiving the necessary attention and importance. Currently, Brazil is one of the three eco-epidemiological hotspots for human cases [1], and ranks first for scientific research on the topic [29–31].

In Presidente Prudente, considered an axis linking endemic to non-endemic regions of São Paulo state, *Lu. longipalpis* was first captured in 2009 in area 3 and area 2 in the northern part of the urban area, near the downtown area. With an appropriate environment not only for blood-meal sources but also vulnerable dogs acquiring the infection, this region is bordered by the Limoeiro stream, with an area of dense forest fragments. Furthermore, there is a large non-urbanized region of abandoned buildings, including slaughterhouses and tanneries. Interestingly, this was also an area with a high concentration of individuals infected in the 2005 dengue fever outbreak. In the entomological survey, from 2009 to 2012, 71.4% of the vectors were captured in areas 2, 3 and 5, in the downtown area. However, from 2013 to 2015, 70% of vectors were captured in areas 1, 4 and 6, located on the outskirts. The sand flies have spread progressively from central to peri-urban neighborhoods (Table 1, Fig. 2). In Dracena, the first endemic focus of VL from RNHA 11 and mesoregion 8, *Lu. longipalpis* was identified in 2003 and spread throughout the city within one year [32, 33]. In São Paulo, it was proposed that the *Lu. longipalpis* sex pheromone (S)-9-methylgermacrene-B population, linked to the expansion of VL in the western region, has a greater vector capacity than the *Lu. longipalpis* sex pheromone cembrene-1, present in other regions of the state [34]. This finding may explain the fast spread of vectors in the urban areas of cities in RNHA 11. How the *Lu. longipalpis* sand flies reached Presidente Prudente and spread is not clear. Although the entomological survey began in 1999, the first sand flies were found in 2009, in area 3 near the downtown. In this setting, there was a large flower shop with production of ornamental trees and fruits trees, where large amounts of organic fertilizer were used. Within about 80 m, there was a chicken coop, two dogs and large amounts of organic matter, where 756 sand flies were found. It is plausible to suggest that larva or sand flies came from VL endemic regions in the surrounding area of Presidente Prudente in the organic matter, vases or materials for the flower shop. From this area, the sand flies spread to the other sites. In 2011, they were found in new sectors of areas 2 and 3, in addition to the initial area (Fig. 2).

**Fig. 8:**
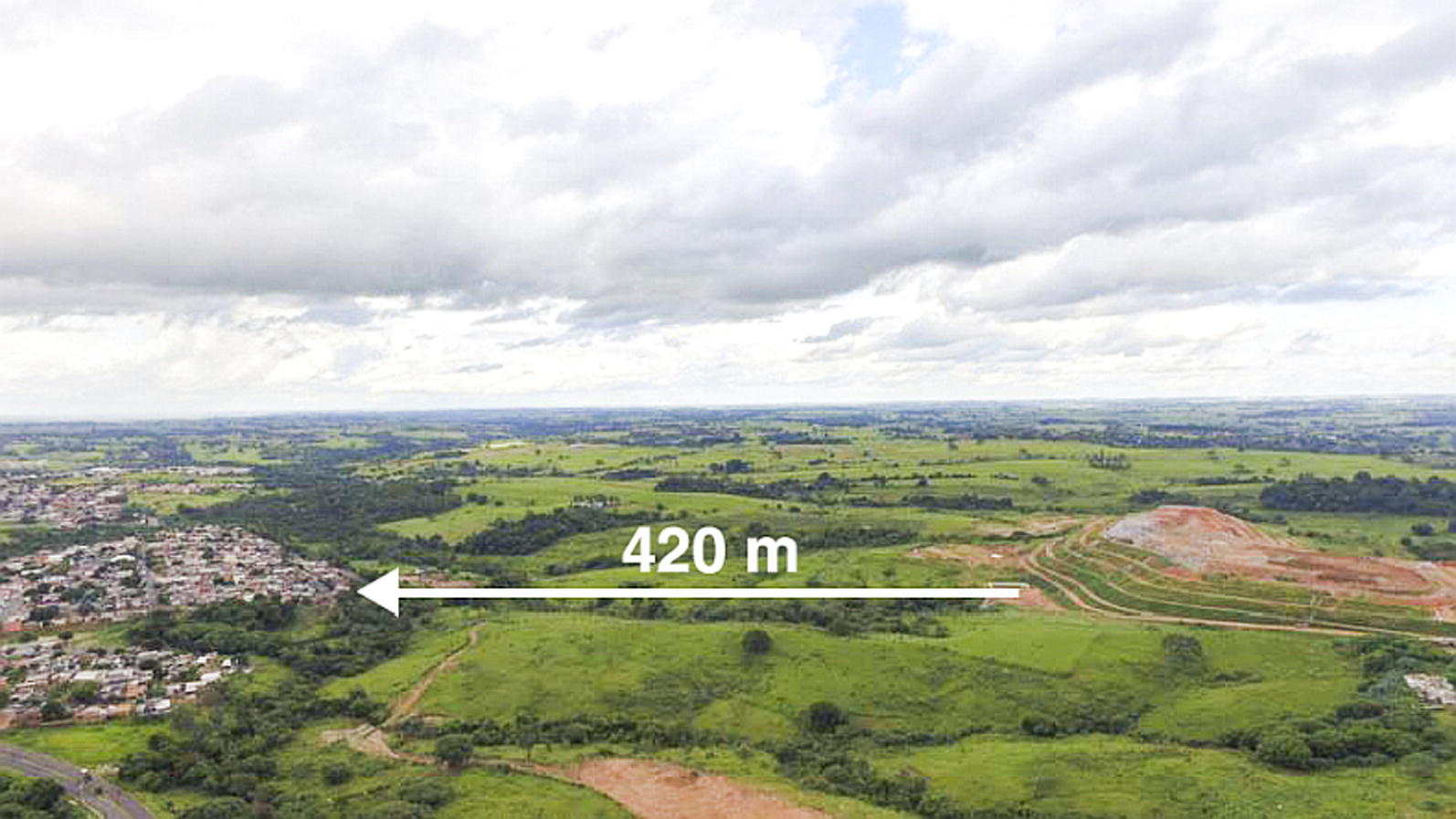
Area 4 in the eastern zone, forest fragments, rural farms and public landfill. Perspective view of Cambuci and José Rota neighborhoods and distance to the urban solid-waste management project (approximately 420 m). Picture taken on December 3, 2017

*Canis familiaris* (domestic dogs) are believed to be the most influential urban reservoir of *Leishmania infantum*, and in urban settings, the transmission cycle of zoonotic VL in dogs is well established. It has been shown that human epidemics of VL are usually preceded by or concomitant with CVL [35–38]. In this study, from 2010 to 2015, 40,309 dogs were serologically screened and 638 (1.6%) were found to be positive for VL by ELISA-IFAT (2010–2011) and DPP-ELISA (2012–2015). This result is lower than that previously found (4.5%; *P* < 0.05) in a cross-sectional study conducted using the same approach from January 2010 to July 2011 in the city of Presidente Prudente and Montalvão district [9]. This difference may be due to the fact that at the beginning of the serological survey, control measures had not yet been implemented. Thus, the number of seropositive dogs tended to be higher than observed later in the survey period. Few studies in São Paulo state have addressed the seroprevalence of CVL, especially with such a large number of dogs. From August 2005 to January 2008, in a study using the same methodology carried out among 12 municipalities of RNHA 11 in the Dracena microregion, 29,995 dogs were analyzed with a seroprevalence of 23.8% [39]. The results were 15.8-fold higher than found in this study, probably because the samples came from municipalities where the disease had been previously established, some of which are considered endemic. In these sites *Lu. longipalpis* was detected in 2003; CVL and HVL have been detected since 2006 [39]. To our knowledge, this is one of the largest serological surveys for screening of CVL carried out in Brazil. Taken together, 70,204 animals were analyzed, representing a significant landscape of the burden of CVL in the western region of São Paulo state. In Belo Horizonte, the capital of Minas Gerais state, during 2007–2009, blood samples from 470,479 dogs were tested and 7.8% were seropositive [16]. It is anecdotally well-known that people from different regions of Presidente Prudente abandon their dogs in areas 2 and 3, where they roam freely in the streets and abandoned areas, becoming a target for *Lu. longipalpis* sand flies. In the two-year analysis, according to the kernel map, the first biennium 2010–2011 showed a higher number of hotspots of dogs seropositive for VL (Fig. 5). We suggest that in these areas, the spatiotemporal presence of the vectors in 2009–2010 resulted in a high concentration of infected dogs in 2010–2011. Area 4 in the eastern zone is also considered a highly vulnerable setting fueled by particular environmental risk factors and high levels of CVL throughout 2010–2015. In this peri-urban area of Presidente Prudente, there is a landfill site (Fig. 8), the Gramado stream, a dense forest fragment, and several irregular solid waste deposits (Fig. 6, circle A). In these settings, wandering dogs, cats and rodents may be the source of blood meals, attracting female sand flies. Worldwide, sand fly density and the peri-urban blood-feeding behavior increase the risk of CVL transmission [14–16]. In area 4, in a spatiotemporal distribution, infected dogs were first found in 2010 and have remained at high levels since then; vectors were found in 2013 and the first human infected with VL in Presidente Prudente was also diagnosed in 2013.

In the last biennium, 2014–2015, a new hotspot of CVL appeared in the southeastern area, area 1. The area is located on the extreme periphery, 10.5 km from downtown. In that region, a large number of households were built 26 years ago in a popular government housing project. On the poor periphery of medium- and large-sized cities in developing countries, particularly in Brazil, lack of sanitation, sites of irregular domestic waste disposal, and increased numbers of stray dogs and cats are harmful risk factors for the dispersion of VL [40–43]. In a study carried out in a VL endemic area of Barra de Guaratiba, Rio de Janeiro state, higher rates of infection occurred in places located on the outskirts where some the houses were bordered by forest fragments [44]. In our spatiotemporal approach, vectors were found in different sectors of area 1 in 2013, the same area that presented high levels of infected dogs in 2014–2015 and where one individual was diagnosed with VL in 2016 and one in 2017. In the downtown area and in the middle- and upper-income residential condominiums areas on the outskirts, fewer seropositive dogs were found. In these settings, dogs are confined to their owner’s house, with pet care services and good health care (Fig. 3, areas B and C). Several measures have been taken to tackle the burden of CVL in Presidente Prudente. According to the CZC, between 2013 and 2017, more than 57,000 domestic dogs were microchipped with identity numbers and tested for the presence of CVL. Because of conflicting results about the role of culling infected dogs in CVL control [45], a lawsuit project prohibiting the euthanasia of dogs and cats without clinical signs of leishmaniasis has been discussed in Presidente Prudente and countrywide.

Nowadays, studies have been carried out on the problem of municipal solid waste, but few discuss irregular deposits in urban settings. It is alarming that 56 irregular solid-waste deposits were found in Presidente Prudente, mainly along the river basins, in vacant household lots. They were found not only on the periphery but also near the downtown area. Unfortunately, although the Mayor’s office environmental coordination team regularly cleans up vacant lots, huge amounts of garbage are repeatedly deposited. There have been intensive local campaigns on posters, leaflets, radio and TV about the role of garbage in the proliferation of vectors for VL and dengue and the increasing number of dogs and humans infected; however, some people have completely ignored them. The correct disposal of waste is one of the components of the Health Vulnerability Index associated with the occurrence of VL [16]. Most of the populations living in endemic areas know that the disease spreads by female sand fly bites. Sand flies are most likely attracted to such sites because of the presence of rodents, dogs, cats and others sources of blood meals [40, 41]. To our knowledge, this is the first description of the association between urban-mapped irregular solid-waste deposits and the spread of VL in Brazil. As a result of violence and insecurity in medium- and large-sized cities in Brazil, high- and middle-class people are moving from neighborhoods near the downtown to middle- and upper-income residential condominiums on the outskirts, which involve new roads, establishing new areas of inhabitants, and progressively extending the limits of the urban perimeter. On the other hand, as a result of exploitation by private entrepreneur groups in land occupation, dozens of popular government housing projects financed by public agencies are also being built on the outskirts. As a consequence, there are large tracts of undeveloped land between neighborhoods and popular village complexes/private condominiums. These are environmentally vulnerable areas for vectors and the spread of CVL (Fig. 3, areas A, B and D). Based on the 2014–2015 biennium, although not statistically significant, clusters of cases of CVL were located near forest fragments, water drainage and irregular disposal of solid waste present within a radius of 1200 m in areas 1 and 4, located on the outskirts of Presidente Prudente (Fig. 6, areas A–C). However, the disease was not limited to the periphery and a hotspot was found near the downtown area (Fig. 6, area B), similar to others cities in São Paulo state and Campo Grande, the capital of Mato Grosso do Sul state [33, 46]. In cities of Teresina, São Luis and Aracaju, in the northeast of Brazil, outbreaks of visceral leishmaniasis were related to the rapid and chaotic urban growth in areas surrounded by slums as a consequence of intensive migratory flow [47, 48]. In Fig. 7, the kernel map was generated in the square locations of the households with positive dogs. When the spatial distribution of CVL cases in the biennium 2014–2015 and the distribution of irregular solid waste deposits mapped in 2015 were analysed, the densities of the CVL distributions were spatially close to most of the densities of the irregular solid waste deposits, but not sufficient to generate a statistical association. Assuming that the points of irregular solid waste deposits are probably constant in their spatial distribution over time, the relationship in 2010–2011 and in 2012–2013 may be representative.

Presidente Prudente can be considered an urban setting with recent transmission, and a few reported cases of HVL have occurred, mainly on the outskirts and associated with places with high rates of CVL. Although they were diagnosed and treated in a reference, public, university tertiary hospital, two of the six patients died a mortality rate of 33.3%, higher than 8.6% in São Paulo state [5]. In new endemic regions, the lack of knowledge of patients and clinicians about the signs and symptoms and the delay in diagnosis and treatment may contribute to an unfavourable prognosis of the disease [11, 49].

One of the main shortcomings when interpreting our data is the fact that we do not know the extent of veterinary follow-up and the number of dogs that progressed to chronic disease and subsequent euthanasia. Although screening and confirmatory tests were applied, the possibility of false-positive or false-negative results in the serological techniques used for dogs cannot be ruled out. Our results have global relevance, because distressing issues, including difficulties in dealing with the control of vectors, the controversial measure of culling infected dogs, the role of the environment, particularly inefficient control of domestic waste, in the spread and maintenance of VL are shared globally, mainly in developing countries.

## Conclusions

In Presidente Prudente, the spatiotemporal occurrence of vectors, CVL and HVL and the presence of environmental risk factors may be taken as a challenge for local and regional authorities. Our findings may be replicated in other new endemic regions, mainly in developing countries, that have not only environmental but also cultural and socio-economic similarities. The results may be useful to help public health authorities in planning prevention and control measures to avoid expansion of CVL and futures outbreaks of VL.

## Data Availability

The data supporting the conclusions of this article are included within the article. The datasets generated and/or analyzed during the present study are not publicly available. Data on the entomology survey were generated within the Regional Entomology Laboratories of SUCEN of Presidente Prudente. Data on CVL were generated within the Center of Zoonosis Control of Presidente Prudente and by the Adolfo Lutz Institute of Presidente Prudente. Data on HVL were obtained from the Municipal Surveillance Epidemiology Center.

## Abbreviations

CRL-CBSALI-PPV: Center of Regional Laboratory of the Adolfo Lutz Institute of Presidente Prudente V
CVL: canine visceral leishmaniasis
CZC: Center for Zoonosis Control
DPP: dual-path platform
HVL: human visceral leishmaniasis
IBGE: Brazilian Institute of Geography and Statistics
IFAT: indirect fluorescent antibodies
RNHA 11: Regional Network for Health Assistance 11
SUCEN: Supervision and Control of Epidemic
VL: visceral leishmaniasis
VLCSP: Visceral Leishmaniasis Control and Surveillance Programme
